# 25-Hydroxyvitamin D Status and Risk for Colorectal Cancer and Type 2 Diabetes Mellitus: A Systematic Review and Meta-Analysis of Epidemiological Studies

**DOI:** 10.3390/ijerph14020127

**Published:** 2017-01-28

**Authors:** Cem Ekmekcioglu, Daniela Haluza, Michael Kundi

**Affiliations:** Department of Environmental Health, Center for Public Health, Medical University of Vienna, Kinderspitalgasse 15, Vienna A-1090, Austria; daniela.haluza@meduniwien.ac.at (D.H.); michael.kundi@meduniwien.ac.at (M.K.)

**Keywords:** vitamin D, 25-hydroxyvitamin D, colorectal cancer, type 2 diabetes mellitus, systematic review, meta-analysis

## Abstract

Epidemiological evidence suggests an association between low vitamin D status and risk for various outcomes including cardiovascular diseases, cancer, and type 2 diabetes mellitus (T2DM). Analyzing serum 25-hydroxyvitamin D [25(OH)D] is the most established means to evaluate an individual’s vitamin D status. However, cutoff values for 25(OH)D insufficiency as well as for optimal 25(OH)D levels are controversial. This systematic review critically summarizes the epidemiological evidence regarding 25(OH)D levels and the risk for colorectal cancer and T2DM. The meta-analytical calculation revealed a pooled relative risk (RR) of 0.62 (CI 0.56–0.70; I^2^ = 14.7%) for colorectal cancer and an RR of 0.66 (CI 0.61–0.73; I^2^ = 38.6%) for T2DM when comparing individuals with the highest category of 25(OH)D with those in the lowest. A dose–response analysis showed an inverse association between 25(OH)D levels and RR for both outcomes up to concentrations of about 55 ng/mL for colorectal cancer and about 65 ng/mL for T2DM. At still higher 25(OH)D levels the RR increases slightly, consistent with a U-shaped association. In conclusion, a higher 25(OH)D status is associated with a lower risk for colorectal cancer and T2DM; however, this advantage is gradually lost as levels increase beyond 50–60 ng/mL.

## 1. Introduction

The major functions of vitamin D are related to calcium and bone metabolism. Many studies in recent years have further postulated an important role of vitamin D in several other physiological systems [1]. For example, vitamin D has been suggested to stimulate insulin secretion and decrease insulin resistance [2] and to also exert anti-cancer activity, including anti-proliferative and anti-inflammatory effects [3].

The established general biomarker for the vitamin D status is 25(OH)D, which reacts to dietary vitamin D intake as well as endogenous vitamin D production [4,5]. Lips [6] proposed to classify vitamin D deficiency according to serum 25(OH)D levels into three stages: severe deficiency (<12.5 nmol/L; <5 ng/mL), moderate deficiency (12.5–25 nmol/L; 5–10 ng/mL), and mild deficiency or insufficiency (25–50 nmol/L; 10–20 ng/mL). The thresholds for severe deficiency and partly also moderate deficiency are more or less accepted by the scientific community. However, the thresholds for insufficiency and repletion or optimal status have been controversial, especially since 2011. At that time the Institute of Medicine (IOM) suggested a minimum serum level of 50 nmol/L (20 ng/mL) as the value at which 97.5% of the vitamin D needs of the population would be covered [7,8]. In contrast to the IOM, the Endocrine Society defined vitamin D deficiency as a 25(OH)D below 20 ng/mL (50 nmol/L) and vitamin D insufficiency as a 25(OH)D of 21–29 ng/mL (52.5–72.5 nmol/L) [9].

The discrepancy between the Endocrine Society and IOM clinical guidelines is the result of different ratings of the effects of vitamin D on bone health [10,11]. For example, a previous review of randomized controlled trials (RCT) and cohort studies by Bischoff-Ferrari et al. [12] suggested that for the endpoints bone mineral density (BMD), lower extremity function, dental health, risk of falls, fractures, and colorectal cancer the most advantageous serum concentrations of 25(OH)D exceeds 75 nmol/L (30 ng/mL), and the best results were achieved at levels between 90 and 100 nmol/L (36–40 ng/mL).

In addition to its widely studied effects on bone and mineral metabolism, low 25(OH)D levels have also been associated with development of T2DM [13], hypertension [14], hyperlipidemia [15], and cardiovascular diseases [16]. Therefore, in addition to relating the 25(OH)D status to bone health, relevant 25(OH)D concentrations should also be discussed by including results on non-bone-related endpoints. For example, results of a cross-sectional study among 239 overweight and obese, sedentary postmenopausal women without T2DM found a threshold effect of 25(OH)D on glucose–insulin metabolism [11]. This observation thus suggests that 25(OH)D ≥~26 ng/mL is associated with normal glucose homeostasis. Furthermore, six meta-analyses of observational studies found an inverse association of blood 25(OH)D levels in relation to the risk of colorectal cancer [17,18,19,20,21,22].

Vitamin D is not only important for bone health, as there is an intensive ongoing debate about 25(OH)D reference values. Thus, this systematic review aims to critically summarize the epidemiological evidence regarding 25(OH)D levels and the risk for colorectal cancer and T2DM. For both outcomes several case-control and cohort studies, which compared risks across strata differing in serum 25(OH)D levels, will be systematically reviewed and analyzed regarding potential dose-dependent effects in order to gain more insight into optimal serum 25(OH)D levels. This systematic review is an update of two previous meta-analyses [23,24], which studied the association between blood 25(OH)D levels and incident type 2 diabetes [23] or colorectal cancer [24], respectively. In their meta-analyses Song et al. [23] found a summary relative risk for type 2 diabetes of 0.62 (95% CI 0.54–0.70) comparing the highest to the lowest category of 25(OH)D levels, and Lee et al. [24] calculated an OR of 0.66 (95% CI, 0.54–0.81) for colorectal cancer when comparing top versus bottom quantiles of circulating 25(OH)D levels.

## 2. Methods

Two researchers (Cem Ekmekcioglu, Daniela Haluza independently performed a systematic literature search in PubMed and Scopus for the two outcomes colorectal cancer and T2DM. The search was an update of two previous meta-analyses on risk ratios for the indicated endpoints in relating to 25(OH)D levels [23,24]. The searches were restricted to cohort and case-control studies, English publications and relative risk estimates over different 25(OH)D categories had to be available with 95% confidence intervals or other indicators of variance.

1. Colorectal cancer: An update of the meta-analysis by Lee et al. [24] was performed on 31 August 2016 using the terms (vitamin D or 25(OH)D or 25-hydroxyvitamin D) and (colorectal cancer or colon cancer or rectal cancer) with studies published after 1 March 2010 (PubMed) or 1 January 2010 (Scopus).
2. T2DM: An update of the meta-analysis by Song et al. [23] was performed on 31 August 2016 using the terms (vitamin D or 25(OH)D or 25-hydroxyvitamin D) and (diabetes or diabetes mellitus) with studies published after 1 January 2012.

Each publication, whether previously included in a meta-analysis or added during the update, was independently assessed by two reviewers (Cem Ekmekcioglu, Michael Kundi) and entered into a database. If several analyses differing in adjustment for covariates were reported, we used those analyses including the greatest number of relevant confounders. In several cases different subgroups were reported; this structure was maintained during abstraction.

For the purpose of this meta-analysis all 25(OH)D levels reported in nmol/L were transformed to ng/mL by applying a conversion factor of 0.40064. As studies differed with respect to the reference category, we recalculated the risk estimates by consistently using the lowest category of 25(OH)D as the reference. We used the fixed effects and the random effects model by DerSimonian and Laird for estimating the overall meta-analytical effect size and 95% confidence intervals. Homogeneity across studies was tested by Cochran’s Q test and expressed as percent deviation from random variation (I^2^). Although some heterogeneity across studies was found there was no indication of distortion of the overall estimates by the few results that were responsible for this heterogeneity. Therefore, we report the results of the fixed effects model (I–V, inverse variance weighting) in the results section. The results of the random effects model, however, are also shown in the graphs.

Two meta-analyses were performed: one for the highest category of 25(OH)D against the lowest and one for the category that was closest to a level of 20–30 ng/mL, if available. Possible small studies bias was assessed by Funnel plots and Egger’s regression. Restricted cubic spline analyses were performed after recalculating point estimates for all categories relative to a reference of 12 ng/mL (as the midpoint of the reference interval). For this recalculation a factor relating the reference category to one with 12 ng/mL served as a midpoint. The relative risk estimate of the common reference to the reported one was obtained by linear interpolation. All analyses were performed using Stata 12.0 (StataCorp, College Station, TX, USA).

## 3. Results

### 3.1. Colorectal Cancer

The literature search in PubMed and Scopus retrieved 224 and 144 articles, respectively. After scanning the titles and abstracts and excluding papers with patients suffering from colorectal adenoma, we selected 21 papers for further evaluation. From those papers, 14 were finally selected after reading the full articles. The reasons for excluding the remaining papers were: (1) no indication of 25(OH)D categories or risk ratios over categories [25,26,27]; (2) inclusion of patients with inflammatory bowel diseases [28]; and (3) the outcome was cancer mortality [29,30,31].

So, in addition to the 10 studies included in the meta-analysis of Lee et al. [24], a total of 24 studies were included in the systematic review [24,32,33,34,35,36,37,38,39,40,41,42,43,44,45,46,47,48,49,50,51,52,53,54]. These are summarized in Table 1. Most of the studies were from the USA, some from Europe, and a few from other regions. According to the multivariate adjusted risk ratios over categories of 25(OH)D, in general higher 25(OH)D was associated with lower risk for colorectal cancer with only a few exceptions [41,44].

**Figure 1:**
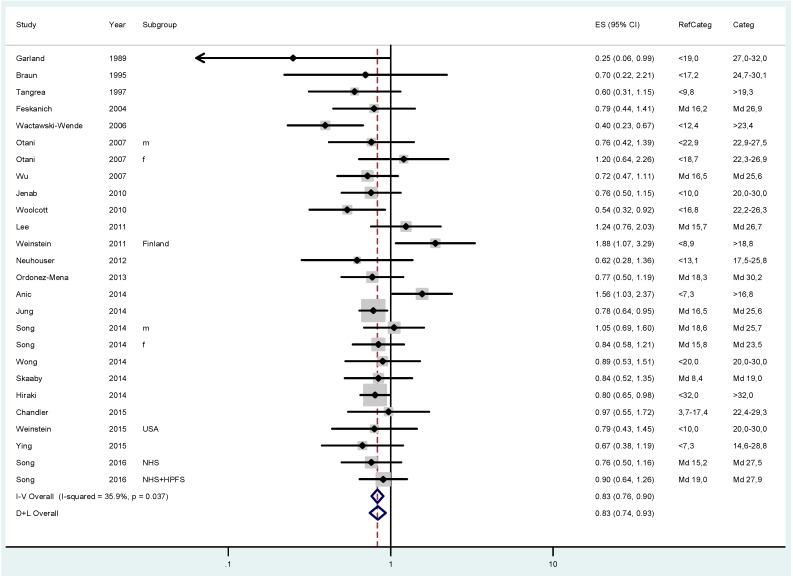
Results of the fixed (I–V) and random-effects (D–L) meta-analysis regarding the effects of 25(OH)D status on colorectal cancer risk in case-control and cohort studies. Participants with 25(OH)D concentrations between approximately 20 and 30 ng/mL (50–75 nmol/L) were compared with those in the lowest 25(OH)D category (RefCateg). The effect sizes (ES) as relative risk estimates and 95% confidence intervals (CI) are shown.

**Figure 2:**
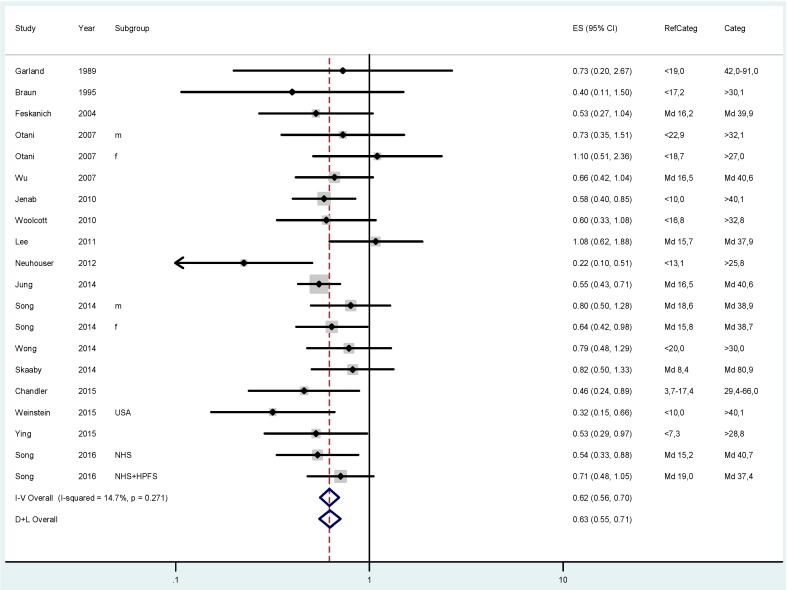
Results of the fixed (I–V) and random-effects (D–L) meta-analysis regarding the effects of 25(OH)D status on colorectal cancer risk in case-control and cohort studies. Participants with 25(OH)D concentrations highest were compared with those in the lowest 25(OH)D category (RefCateg). The effect sizes (ES) as relative risk estimates and 95% confidence intervals (CI) are shown.

The pooled relative risk (RR) for colorectal cancer was 0.83 (95% CI 0.76–0.90; I^2^ = 35.9%) when comparing participants with approximately 20–30 ng/mL 25(OH)D status with those in the lowest category (Figure 1). The risk further dropped to an overall RR of 0.62 (CI 0.56–0.70; I^2^ = 14.7%) when comparing individuals with the highest vitamin D status with those in the lowest group (Figure 2).

**Figure 3:**
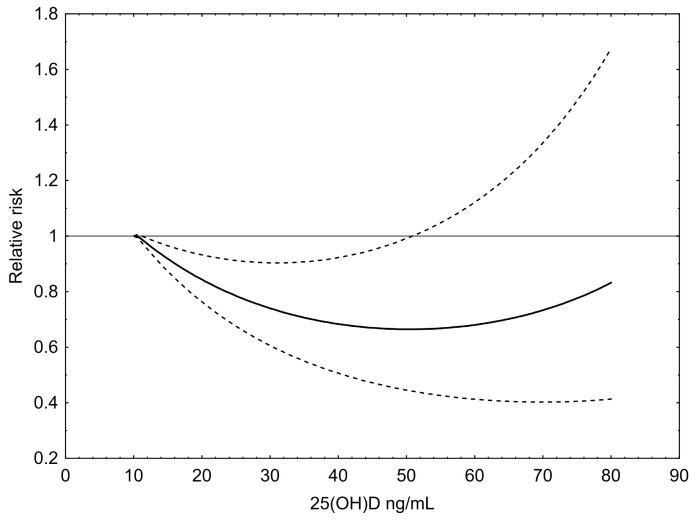
Dose–response relationship between 25(OH)D concentrations and the relative risk for colorectal cancer. Results of restricted cubic splines analysis of relative risks standardized to a common reference category with 12 ng/mL 25(OH)D as midpoint and inverse variance weights. Dashed lines indicate 95% confidence interval.

For the graphical dose response analysis a total of 111 risk estimates for colorectal cancer were included (Figure 3). An inverse, moderately U-shaped association between 25(OH)D levels and colorectal cancer risk can be seen. The nadir of the curve lies at 55 ng/mL (~137 nmol/L) meaning that at this 25(OH)D level the RR of colorectal cancer was lowest with a RR of approximately 0.65 compared to 12 ng/mL, which was set as the reference. However, the RR increases at very high levels of 25(OH)D up to 80 ng/mL, although the RR was still lower at the right end of the curve than at 12 ng/mL.

Heterogeneity across studies was moderate and sensitivity analyses leaving out one study in turn revealed risk estimates between 0.81 and 0.84 for the test category of approximately 20–30 ng/mL and risk estimates between 0.61 and 0.64 for the highest 25(OH)D category against the lowest. Heterogeneity was due to two studies only [41,44], both from Finland and with low levels in the highest 25(OH)D category.

### 3.2. Type 2 Diabetes Mellitus 

The literature search in PubMed and Scopus retrieved 1136 and 554 articles, respectively. After scanning the titles and abstracts, 21 papers were extracted for further evaluation. From those papers, 12 were finally selected after reading the full reports. The remaining papers [55,56,57,58,59,60,61,62,63,64] were excluded, especially because no risk ratios or no 25(OH)D categories were available for calculation, children or prediabetic patients were included, or diabetic complications were evaluated. So, in addition to the 15 publications included in the meta-analysis of Song et al. [23], and a paper from 2007 [65], a total of 28 publications were included in the systematic review [55,65,66,67,68,69,70,71,72,73,74,75,76,77,78,79,80,81,82,83,84,85,86,87,88,89,90,91]. These are summarized in Table 2. Most of the studies analyzed T2DM as the outcome variable, with a few considering patients with insulin-requiring diabetes [81]. The origin of the participants was mainly from European countries, partly also from the USA, and a few from other locations. When analyzing the multivariate adjusted risk ratios over 25(OH)D categories it can be observed that in general higher 25(OH)D were associated with lower risk for T2DM with only a few non-significant exceptions [73,90].

**Figure 4:**
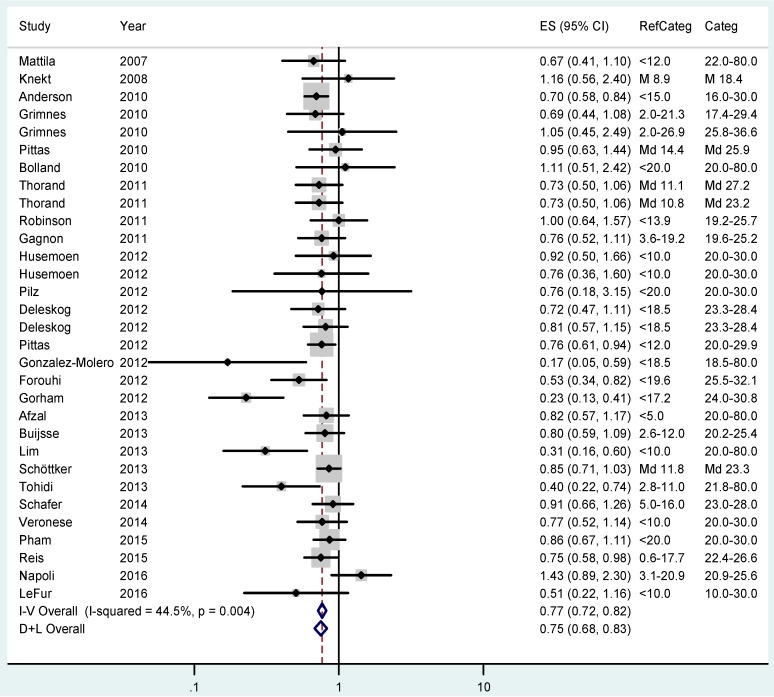
Results of the fixed (I–V) and random-effects (D–L) meta-analysis regarding the effects of 25(OH)D status on type 2 diabetes mellitus risk in case-control and cohort studies. Participants with 25(OH)D concentrations between approximately 20–30 ng/mL (50–75 nmol/L) were compared with those in the lowest 25(OH)D category (RefCateg). The effect sizes (ES) as relative risk estimates and 95% confidence intervals (CI) are shown.

**Figure 5:**
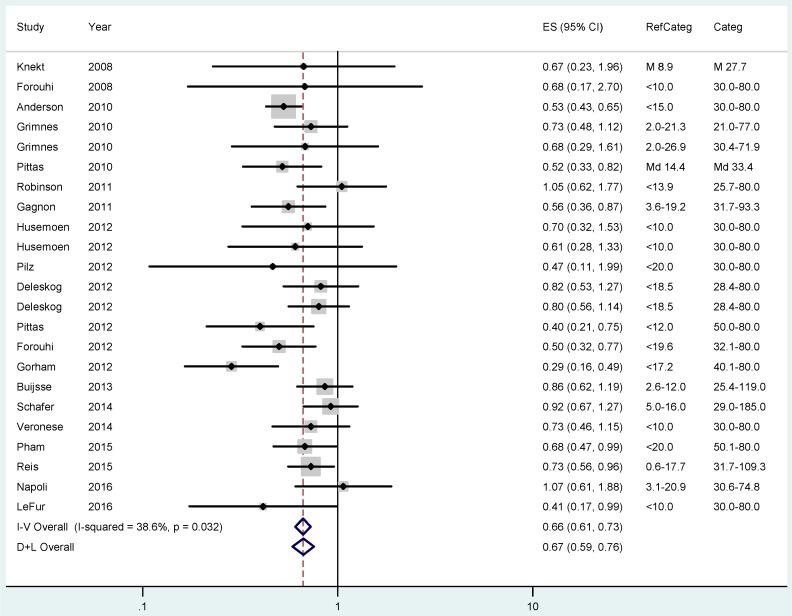
Results of the fixed (I–V) and random-effects (D–L) meta-analysis regarding the effects of 25(OH)D status on type 2 diabetes mellitus risk in case-control and cohort studies. Participants with the highest 25(OH)D concentrations were compared with those in the lowest 25(OH)D category (RefCateg). The effect sizes (ES) as relative risk estimates and 95% confidence intervals (CI) are shown.

The pooled RR for T2DM was 0.77 (95% CI 0.72–0.82; I^2^ = 44.5%) when comparing participants with approximately 20–30 ng/mL 25(OH)D status with those in the lowest category (Figure 4). The risk further declines to an overall RR of 0.66 (CI 0.61–0.73; I^2^ = 38.6%) when comparing individuals with the highest vitamin D status with those in the lowest group (Figure 5).

**Figure 6:**
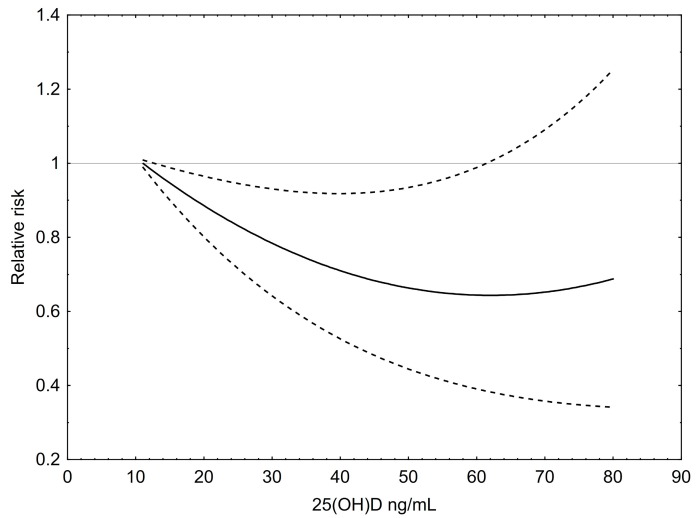
Dose–response relationship between 25(OH)D concentrations and the relative risk for type 2 diabetes mellitus. Results of restricted cubic splines analysis of relative risks standardized to a common reference category with 12 ng/mL 25(OH)D as midpoint and inverse variance weights. Dashed lines indicate 95% confidence interval.

For the illustrated dose–response analysis a total of 119 risk estimates for T2DM were included (Figure 6). Similar to the outcome for colorectal cancer, an inverse, slightly U-shaped association between 25(OH)D levels and T2DM risk was found. The nadir of the curve lies at 65 ng/mL (~162 nmol/L), a concentration that is associated with the lowest RR of approximately 0.65 compared to 12 ng/mL, which was set at 1. However, the curve also rises to some extent at its right end, meaning that the RR starts to increases at very high levels of 25(OH)D, but still remains below 1.

Heterogeneity across the studies was higher than for colorectal cancer but sensitivity analyses leaving out one study in turn still revealed a narrow range of resulting risk estimates between 0.76 and 0.78 for the test category of approximately 20–30 ng/mL and risk estimates between 0.65 and 0.70 for the highest 25(OH)D category against the lowest. Heterogeneity was mainly due to two studies [81,90]: one study [81] had insulin-dependent diabetes as the endpoint and found a greater than average reduction of risk by increasing levels of 25(OH)D; the other [90] was a study in elderly men that could not establish any relationship to development of diabetes. Omitting these studies had no effect on the meta-analytical risk estimate, but removed the residual heterogeneity to a non-significant 10.4%.

## 4. Discussion

Vitamin D deficiency or insufficiency has become (and possibly was for several decades) a public health problem worldwide [93,94,95,96]. Mithal et al. [93], for example, described in their global report that most populations do not have a satisfactory vitamin D status and especially people with other risk factors and elderly people are risk groups for vitamin D deficiency. A systematic review of 195 studies, including more than 168000 participants from 44 countries, showed considerable variations in mean 25(OH)D values. Approximately 37% of studies reported mean values below 20 ng/mL [95].

A vast number of publications and meta-analyses reported associations between vitamin D and various health outcomes involving nearly all organ systems (summarized in [97,98]). However, although there is an extensive literature available, a consensus on optimal intakes of vitamin D and especially reference levels of 25(OH)D is missing so far [99]. This is at least partly due to the lack of randomized controlled trials of vitamin D supplementation for endpoints other than bone health [99,100].

Regarding bone health, a meta-analysis of 12 double-blind RCTs for non-vertebral fractures (*n* = 42,279) and eight RCTs for hip fractures (*n* = 40,886) established that the fracture-protecting effects of vitamin D are dose-dependent and increase with higher serum 25(OH)D levels [101]. Furthermore, it was also shown that fall prevention occurred with 25(OH)D levels of 60 nmol/L (24 ng/mL) up to 95 nmol/L (38 ng/mL) [102], while ~75–112 nmol/L (~30–45 ng/mL) was necessary for non-vertebral fracture prevention [101]. Additionally, another more recent pooled analysis by Bischoff-Ferrari et al. [103] from 11 double-blind RCTs of oral vitamin D supplementation showed that, compared to <30 nmol/L (<12 ng/mL), individuals with 25(OH)D levels of at least 61 nmol/L (24 ng/mL) had a 37% reduction of hip fracture risk and 31% reduction of any non-vertebral fracture risk. Persons having baseline 25(OH)D levels of at least 43 nmol/L (17 ng/mL) already showed a significantly reduced risk for any non-vertebral fracture compared to those with <30 nmol/L (<12 ng/mL). Related to these data, it was suggested that > 50 nmol/L (> 20 ng/mL) is a minimum general level, whereas more than 60 nmol/L (24 ng/mL) may be required for optimal (bone) health benefits.

In addition to bone health, an increased overall mortality risk was shown in a large German population-based cohort with decreasing 25(OH)D levels less than ~75 nmol/L (~30 ng/mL) [104]. These results were confirmed in a recent meta-analysis also showing that serum 25(OH)D levels less than or equal to 75 nmol/L (30 ng/mL) were associated with higher all-cause mortality compared with levels >75 nmol/L [105].

In addition to mortality, reduced serum 25(OH)D was shown to correlate with insulin resistance, obesity, aberrant phasing of insulin responses to glucose loading, glucose intolerance, fasting hyperglycemia, or also T2DM [106,107,108]. In a recent cross-sectional study in community-dwelling men aged 70 and older it was shown that poor health, self-reported diabetes mellitus and hyperglycemia, depression, muscle weakness and poor balance, and also all-cause mortality were associated with serum 25(OH)D levels lower than 50 nmol/L (20 ng/mL). However the findings also suggested that for a wide range of health conditions, including falls and mortality, the optimum range of 25(OH)D could be between 50.0 and 74.9 nmol/L (20–~30 ng/mL), with no more benefit for 25(OH)D levels of 75 nmol/L (30 ng/mL) or greater [109].

In a meta-analysis by Mitri et al. [13], which included five cohorts from four studies, it was found that the risk of getting T2DM was 43% lower when 25(OH)D was >25 ng/mL (62 nmol/L) compared to 14 ng/mL (35 nmol/L). Another meta-analysis by Forouhi et al. [80] of 11 prospective studies showed a combined RR of 0.59 (95% CI 0.52–0.67) for T2DM when comparing the highest with lowest quartile of 25(OH)D. In addition, the meta-analysis by Song et al. [23], which included 21 prospective studies, calculated a RR of 0.62 for the highest versus the lowest categories of 25(OH)D.

Our results of RR reduction for T2DM are in line with these meta-analyses, also showing a RR reduction of more than 50% (RR 0.66; 95% CI 0.61–0.73) for the highest vs. lowest 25(OH)D categories. Furthermore, Song et al. [23] showed in a spline regression model in their meta-analysis of 18 prospective studies that higher 25(OH)D levels were inversely associated with a lower diabetes risk up to levels of 160 nmol/L (64 ng/mL) [23]. However, evidence for the protective effects of 25(OH)D levels above 100 nmol/L (40 ng/mL) was weak in their meta-analysis because of only a few studies available in this concentration range. Our study also found an inverse association between 25(OH) levels of up to approximately 65 ng/mL (~162 nmol/L) and RR for T2DM. However, at higher levels the RR approaches a U-shaped association. Some papers have described a U-shaped relationship between 25(OH)D concentrations and various health outcomes (reviewed in Grant et. al. [110]). This may be due to a real harmful effect of high 25(OH)D levels on some disease outcomes or may be due to chance or some confounding factors, such as late supplementation with vitamin D with only small or no effects on disease progression. Also, differences in analytical methods may be a reason for the U-shaped relationships [110].

In a recent umbrella review of meta-analyses it was concluded that suggestive evidence exists for a correlation between high vitamin D concentrations and low risk of, for example, non-vertebral fractures, cardiovascular disease, hypertension, stroke, depression, prevalence of metabolic syndrome, T2DM, and colorectal cancer [97].

Regarding the latter outcome, our results are in line with six previous (dose–response) meta-analysis [17,18,19,20,21,22] also showing an inverse relationship between 25(OH)D levels and risk for colorectal cancer. From these meta-analyses Ma et al. [20], including 2630 cases, found that a 10 ng/mL (25 nmol/L) increment in blood 25(OH)D level reduced the RR by 0.74; Gandini et al. [21] found a risk reduction for a 10 ng/mL increase in serum 25(OH)D by 0.85; and Chung et al. [22] calculated for each 10 nmol/L (4 ng/mL) increase in blood 25(OH)D concentration a pooled adjusted odds ratio of 0.94. Gorham et al. considered five studies and reported that a serum level 25(OH)D of ≥33 ng/mL (83 nmol/L) was associated with a 50% lower risk of colorectal cancer incidence, compared with <12 ng/mL (30 nmol/L) [17]. Grant included 10 datasets with a total of 2883 incident cases of colorectal cancer and found an approximately 50% lower risk at 60 nmol/L (24 ng/mL) 25(OH)D vs. 15 nmol/L (6 ng/mL), with higher levels up to 110 nmol/L (44 ng/mL) further reducing the risk [19].

In line with these meta-analyses, we also calculated a reduced RR of 62% in the highest vs. lowest category of 25(OH)D concentrations. However, similar to T2DM, we also found a moderate rise in relative risk at high levels of 25(OH)D, suggesting a U-shaped association. Only a small number of studies (four for each outcome) were available that included 25(OH)D levels exceeding 40 to 50 ng/mL (100 to 125 nmol/L). Hence the small increase of the RR for colorectal cancer and T2DM at higher levels is only weakly supported by evidence.

In general, most of the observational studies included in our systematic review showed a lower risk for colorectal cancer with increasing 25(OH)D levels (Table 1). However, two papers calculated a rather increased risk [41,44]. The reason might be that the data from these studies were derived from Finnish male smokers, where low UVB levels and especially smoking might have influenced the results. For example, ingredients of cigarette smoke were shown to alter the physiological response to 25(OH)D status [44,111]. It should also be mentioned that the 25(OH)D levels in these studies were very low, with averages of 12 ng/mL [41] and 11 ng/mL in winter and 15.5 ng/mL in summer [44]. Only 25% of participants had 25(OH)D levels exceeding 20 ng/mL [41], or exceeding 21 ng/mL in summer and 17 ng/mL in winter, respectively [44]. In a population with such low levels those with rare higher concentrations may constitute a specific group with confounding features.

In addition to the statements of the IOM and the Endocrine Society [7,9], other recommendations regarding normal/optimal 25(OH)D levels have been published. For example, a recent position paper from Australia and New Zealand states that 25(OH)D levels of 50 nmol/L (20 ng/mL) or greater at the end of winter (and 10–20 nmol/L higher at the end of summer, to allow for seasonal decrease) are required for optimal musculoskeletal health [112]. Workshop participants of the NutriProfiel project from Netherlands concluded that there is sufficient evidence to define 50 nmol/L (20 ng/mL) to 75 nmol/L (30 ng/mL) as the optimal range of 25(OH)D for people 5–64 years of age and 75 nmol/L (30 ng/mL) to 100 nmol/L (40 ng/mL) for those >65 years [100].

Physiologically, vitamin D deficiency would result in reduced intestinal absorption of calcium, which, in turn, would raise parathyroid hormone (PTH) levels. On the other hand, adequate vitamin D status suppresses PTH levels. Therefore, normal 25(OH)D values can also be related to the suppression of PTH. Chapuy et al., for example, were among the first to show that PTH levels are held at a stable plateau of 36 pg/mL as long as serum 25(OH)D values are higher than 78 nmol/L (31 ng/mL) [113].

A major well-known limitation from observational studies is that it is not possible to deduce causal relations from the results, e.g. in our case between the vitamin D status and disease outcomes. Causal effects cannot be excluded, but only associations can be derived from the data, which to some extent limit the significance of former epidemiological studies and also this meta-analysis. For example, a summary of interventional studies did not reveal an effect of vitamin D supplementation on disease occurrence, with only a slight relative risk reduction of all-cause mortality [98].

Several confounders may have influenced the results of the included studies. For T2DM, the risk increases especially with a high body mass index (BMI), low physical activity but also smoking. Regarding the risk for colorectal cancer, in addition to BMI, physical activity, smoking, and alcohol intake, family history and eating habits, like intake of fruits and vegetables or red meat, may also have especially affected the results. However, most of the included studies controlled for the major confounders. Still, inflammatory processes, which are prevalent in various conditions, may also affect 25(OH)D levels, and could therefore limit the interpretation and conclusions from the studies, as postulated in a recent review by Autier et al. [98].

Finally, it should also be considered that single 25(OH)D measurements might be prone to errors and that there seem to be major inter-personal variations in the increase and maximum level of 25(OH)D induced by UVB, as shown in a recent paper by Datta et al. [114].

## 5. Conclusions

Our systematic review and dose–response analysis showed an inverse association between levels of 25(OH)D up to 50–60 ng/mL and RR reduction for colorectal cancer and T2DM. Regarding these two outcomes, the data suggest that optimal 25(OH)D levels clearly lie above 30 ng/mL (75 nmol/L) with a large safety margin. The U-shaped association at high 25(OH)D levels can be possibly explained by a true side effect of nutrients or may be due to confounding factors or analytical biases, which should be addressed in future studies.

## Figures and Tables

**Table 1:** Summary of observational studies for 25(OH)D status and risk for colorectal cancer included in the systematic review.

Reference	Study Design	Population	Country	Participants	Categories	OR/HR
					ng/mL *	(95% CI), Multivariate Adjusted Data were Used
Garland 1989 [32]	Prospective study	Washington County	USA	cc cases: 34 controls: 67	1: 4–19	1: Ref
					2: 20–26	2:0.48
					3: 27–32	3:0.25
					4: 33–41	4:0.21
					5: 42–91	5:0.73
Braun 1995 [33]	Case control study	Washington County	USA	cc cases: 57 controls: 114	1: <17.2	1: Ref
					2: 17.2–20.6	2: 0.3 (0.1–1.0)
					3: 20.7–24.6	3: 0.5 (0.2–1.5)
					4: 24.7–30.1	4: 0.7 (0.2–2.0)
					5: >30.1	5: 0.4 (0.1–1.4)
Tangrea 1997 [34]	Nested case-control study	Alpha-Tocopherol, Beta-Carotene Cancer Prevention Study (ATBC Study)	Finland	crc cases: 146 controls: 290	1: ≤9.8	1: Ref
					2: >9.8–≤13.9	2: 0.7 (0.4–1.3)
					3: >13.9–≤19.3	3: 0.8 (0.4–1.3)
					4: >19.3	4: 0.6 (0.3–1.1)
Feskanich 2004 [35]	Nested case-control study	Nurses’ Health Study	USA	crc cases: 193 controls: 383	1: 16.15 (median)	1: Ref
					2: 22.2 (median)	2: 0.93 (0.53–1.63)
					3: 26.85 (median)	3: 0.79 (0.44–1.40)
					4: 31.2 (median)	4: 0.58 (0.31–1.07)
					5: 39.9 (median)	5: 0.53 (0.27–1.04)
Wactawski-Wende 2006 [36]	Nested case-control study	Women’s Health Initiative (WHI)	USA	invasive crc cases: 322 (VitD Suppl.: 168, Placebo: 154)	1: ≥23.4	1: Ref
					2: 16.9–23.4	2: 1.96(1.18–3.24)
					3: 12.4–16.9	3: 1.95 (1.18–3.24)
					4: ≤12.4	4: 2.53 (1.49–4.32)
Otani 2007 [37]	Nested case-control study	Japan Public Health Center-based Prospective Study	Japan	crc cases: 375 (m = 196, f = 179) controls: 750 (m = 392, f = 358)	1: m: <22.9	1: m: Ref
					f:<18.7	f: Ref
					2: m: 22.9–27.5	2: m: 0.76 (0.42–1.4)
					f: <18.7–22.2	f: 1.0 (0.55–1.9)
					3: m: <27.6–32.0	3: m: 0.76 (0.39–1.5)
					f: <22.3–26.9	f: 1.2 (0.65–2.3)
					4: m:32.1 +	4: m: 0.73 (0.35–1.5)
					f:27.0 +	f: 1.1 (0.50–2.3)
Wu 2007 [38]	Nested case-control study	Health Professionals Follow-up Study (HPFS) + also NHS	USA	crc cases: 179 controls: 356	1: 16.5 (median)	1: Ref
					2: 23.2 (median)	2: 0.94 (0.63 to 1.39)
					3: 25.6 (median)	3: 0.72 (0.47 to 1.11)
					4: 31.9 (median)	4: 0.53 (0.34 to 0.84)
					5: 40.6 (median)	5: 0.66 (0.42 to 1.05)
Jenab 2010 [39]	Nested case-control study	EPIC study participants	10 European countries	crc cases: 1248 controls: 1248	1: <10.0	1: 1.32 (0.87 to 2.1)
					2: ≥10.0 to <20.0	2: 1.28 (1.05 to 1.56)
					3: ≥20.0 to <30.0	3: Ref
					4: ≥30.0 to 40.1	4: 0.88 (0.68–1.13)
					5: ≥40.1	5: 0.77 (0.56 to 1.06)
Woolcott 2010 [40]	Nested case-control study	Multiethnic Cohort Study	USA	crc cases: 229 controls: 434	1: <16.8	1: Ref
					2: 16.8 to <22.2	2: 0.63 (0.37–1.08)
					3: 22.2 to <26.3	3: 0.54 (0.32–0.93)
					4: 26.3 to <32.8	4: 0.62 (0.36–1.07)
					5: ≥32.8	5: 0.60 (0.33–1.07)
Lee 2011 [24]	Nested case-control study	Physicians’ Health Study	USA	crc cases: 229 controls: 389	1: 15.7 (median)	1: Ref
					2: 22.3 (median)	2: 0.71 (0.42–1.21)
					3: 26.7 (median)	3: 1.24 (0.76–2.04)
					4: 37.9 (median)	4: 1.08 (0.62–1.87)
Weinstein 2011 [41]	Prospective case-control study	Alpha-Tocopherol, Beta-Carotene Cancer Prevention Study	Finland	crc cases: 428 controls: 428	1: <10.0 (a priori defined cut-off)	1: 0.68 (0.45, 1.03)
					2: 10.0–<15.0	2: 0.78 (0.51, 1.20)
					3: 15.0–<20.0	3: 0.78 (0.49, 1.25)
					4: 20.0–<30.0	4: Ref
					5: ≥30.0	5: 1.0 (0.49, 2.03)
Neuhouser 2012 [42]	Nested case-control study	Women’s Health Initiative Calcium and Vitamin D Clinical Trial	USA	crc cases: 310 controls: 310	1: <13.1	1: 4.45 (1.96, 10.10)
					2: 13.1–<17.5	2: 1.51 (0.72, 3.14)
					3: 17.5–<25.8	3: 2.76 (1.30, 5.89)
					4: ≥25.8	4: Ref
Ordonez-Mena 2013 [43]	cohort study	ESTHER study (Saarland)	Germany	N = 9482 crc cases: 136	1: 12.5 (median)	1: 1.02 (0.68–1.53)
					2: 18.3 (median)	2: Ref
					3: 18.3 (median)	3: Ref
					4: 30.2 (median)	4: 0.77 (0.50–1.20)
Anic 2014 [44]	Nested case-control study	Alpha-Tocopherol, Beta-Carotene Cancer Prevention Study, (ATBC-Study, 1994, Finland)	Finland	crc cases: 416 controls: 416	1: ≤7.3 (winter) ≤10.8 (summer)	1: Ref
					2: >7.3–≤10.8 (winter) >10.8–≤15.5 (summer)	2: 1.05 (0.70, 1.58)
					3: >10.8–≤16.8 (winter)	3: 1.28 (0.85, 1.92)
					>15.5–≤21.4 (summer)	
					4: >16.8 (winter)	4: 1.56 (1.02, 2.36)
					>21.4 (summer)	
Hiraki 2014 [45]	Nested case-control study	Nurses’ Health Study (NHS), the Health Professionals Follow-up Study (HPFS), and the Physicians’ Health Study (PHS)	USA	3 cohorts, total: crc cases: 881 controls: 1556	1: <322: ≥32	Meta-analysis of 3 cohorts
						1: Ref
						2: 0.80 (0.62, 1.02)
						3: 0.67 (0.52, 0.86)
						4: 0.63 (0.48, 0.82)
Jung 2014 [46]	Nested case study	Nurses’ Health Study (1976) and the Health Professionals’ Follow-up Study (1986)	USA	crc cases: 1059	1: 16.5 (median)	1: Ref
					2: 23.2 (median)	2: 0.78 (0.64–0.94)
					3: 25.6 (median)	3: 0.78 (0.64–0.95)
					4: 31.9 (median)	4: 0.67 (0.54–0.83)
					5: 40.6 (median)	5: 0.55 (0.43–0.71)
Skaaby 2014 [47]	cohort studies	Monica10 study, Inter99 study, and Health2006 study [all Copenhagen County]	Denmark	*N* = 11,119 crc cases: 141	1: 8.4 (median)	1: Ref
					2: 19.0 (median)	2: 0.84 (0.52–1.35)
					3: 35.6 (median)	3: 1.04 (0.66–1.64)
					4: 80.9 (median)	4: 0.82 (0.51–1.35)
Song 2014 [48]	Nested case-control study	Nurses’ Health Study and Health Professionals Follow-up Study	USA	crc cases: 615 controls: 1209	1: m: 18.6 (median)	1: m: Ref
					f: 15.8 (median)	f: Ref
					2: m: 25.7 (median)	2: m: 1.05 (0.69–1.61)
					f: 23.5 (median)	f: 0.84 (0.58–1.20)
					3: m: 31.3 (median)	3: m:0.80 (0.51–1.26)
					f: 29.2 (median)	f: 0.67 (0.45–0.98)
					4: m: 38.9 (median)	4: m: 0.80 (0.50–1.28)
					f: 38.7 (median)	f: 0.64 (0.42–0.98)
Wong 2014 [49]	Prospective cohort study	Health in Men Study (HIMS)	Australia	crc cases: 102 no crc: 3614	1: < 20.0	1: 1.12 (0.64–1.84)
					2: 20.0–30.0	2: Ref
					3: > 30.0	3: 0.88 (0.55–1.40)
Chandler 2015 [50]	Nested case-control study	Women’s Health Study (WHS)	USA	crc cases: 274 controls: 274	1: 3.7–17.4	1: Ref
					2: 17.5–22.3	2: 0.84 (0.50–1.42)
					3: 22.4–29.3	3: 0.97 (0.55–1.73)
					4: 29.4–66.0	4: 0.46 (0.24–0.89)
Weinstein 2015 [51]	Nested case-control study	Prostate, Lung, Colorectal and Ovarian Cancer Screening Trial (PLCO)	USA	crc cases: 476 controls: 476	1: < 10.0	1: 1.26 (0.69–2.30)
					2: 10.0–<15.0	2: 1.19 (0.78–1.83)
					3: 15.0–<20.0	3: 1.32 (0.90–1.94)
					4: 20.0–<30.0	4: Ref
					5: 30.0–<40.1	5: 0.87 (0.58–1.33)
					6: ≥40.1	6: 0.40 (0.17–0.92)
Ying 2015 [52]	Case-control study	Health assessment cohort population in Nanjing First Hospital	China	crc cases: 212 controls: 212	1: <7.29	1: Ref
					2: 7.29–<14.61	2: 0.62 (0.35–1.12)
					3: 14.61–<28.84	3: 0.67 (0.38–1.20)
					4: ≥28.84	4: 0.53 (0.29–0.98)
Song 2016 [54]	Nested case-control study	Nurses’ Health Study and Health Professionals Follow-up Study	USA	crc cases: 318 controls: 624	1: 19.0 (median)	1: Ref
					2: 27.9 (median)	2: 0.90 (0.64 to 1.25)
					3: 37.4 (median)	3: 0.71 (0.48 to 1.05)
Song 2016 [53]	Case-control study	Nurses’ Health Study	USA	crc cases: 378 controls: 689	1: 15.2 (median)	1: Ref
					2: 22.4 (median)	2: 0.88 (0.59–1.32)
					3: 27.5 (median)	3: 0.76 (0.50–1.16)
					4: 31.9 (median)	4: 0.77 (0.50–1.18)
					5: 40.7 (median)	5: 0.54 (0.33–0.87)

cc: colon cancer, crc: colorectal cancer, m: male, f: female, Ref: reference category; * Conversion Factor: nmol/L = ng/mL × 2.496.

**Table 2:** Summary of observational studies for 25(OH)D status and risk for (type 2) diabetes mellitus included in the systematic review.

Reference	Study Design	Population	Country	Participants	Categories	OR/HR
					ng/mL *	(95% CI),
						multivariate adjusted data were used
Mattila 2007 [65]	case-cohort study	Mini-Finland Health Survey	Finland	*N* = 4097	1: <12.0	1: Ref
					2: 12.0–16.4	2: 1.10 (0.75–1.61)
					3: 16.8–22.0	3: 0.80 (0.51–1.25)
					4: >22.0	4: 0.67 (0.41–1.11)
Knekt 2008 [68]	nested case-control study	Finnish Mobile Clinic Health Examination Survey, Mini-Finland Health Survey	Finland	cases: 403 controls: 961	1: 8.9	1: Ref
					2: 13.9	2: 1.07 (0.55–2.05)
					3: 18.4	3: 1.16 (0.56–2.40)
					4: 27.7	4: 0.67 (0.23–1.96)
Anderson 2010 [66]	cohort study	Intermountain Healthcare Population	USA	*N* = 41504	1: ≤152: 16–303: >30	Very Low vs. Normal
						(≤15 vs >30 ng/mL):
						HR (adjusted): 1.89 (1.54–2.33)
						Low vs. Normal
						(16–30 vs >30 ng/mL):
						HR (adjusted): 1.32 (1.12–1.56)
Bolland 2010 [72]	cohort study	Community-dwelling, postmenopausal women	New Zealand	*N* = 1471	1: <20.0	1: HR: 0.9 (0.4, 1.9)
					2: ≥20.0	
Grimnes 2010 [69]	cohort study	Tromsø Study	Norway	*N* = 4157 non-smokers *N* = 1962 smokers	non-smokers:	non-smokers:
					1: 2.0–21.3	1: 1.37 (0.89–2.10)
					2: 13.9–25.0	2: 1.27 (0.82–1.97)
					3: 17.4–29.4	3: 0.94 (0.59–1.51)
					4: 21.0–77.0	4: Ref
					smokers:	smokers:
					1: 2.0–26.9	1: 1.47 (0.62–3.48)
					2: 21.2–31.8	2: 1.76 (0.76–4.05)
					3: 25.8–36.6	3: 1.55 (0.66–3.64)
					4: 30.4–71.9	4: Ref
Pittas 2010 [70]	nested case-control study	Nurses’ Health Study (NHS)	USA	cases: 608 controls: 559	Median values	1: Ref2: 1.09 (0.74–1.61)3: 0.95 (0.63–1.45)4: 0.52 (0.33–0.83)
					1: 14.4	
					2: 20.8	
					3: 25.9	
					4: 33.4	
Gagnon 2011 [67]	prospective study	Australian Diabetes, Obesity and Lifestyle Study	Australia	*N* = 5200	1: 3.6–19.2	1: Ref
					2: 19.6–25.2	2: 0.83 (0.56–1.22)
					3: 25.6–31.3	3: 0.48 (0.31–0.76)
					4: 31.7–93.3	4: 0.68 (0.43–1.07)
Robinson 2011 [73]	nested case-control study	Women’s Health Initiative (WHI) Clinical Trials and Observational Study	USA	*N* = 5140 (postmenopausal)	1: <13.9	1: Ref
					2: 13.9–19.2	2: 1.25 (0.78–1.99)
					3: 19.2–25.7	3: 1.00 (0.64–1.57)
					4: >25.7	4: 1.05 (0.62–1.76)
Thorand 2011 [71]	case-cohort study	Monitoring of Trends and Determinants in Cardiovascular Disease (MONICA)/Cooperative Health Research in the Region of Augsburg (KORA)	Germany	cases: 416 non-cases: 1267	men (median):	1: Ref (model 3)2: 0.85 (0.61–1.17)3: 0.73 (0.50–1.05)
					1: 11.1	
					2: 17.6	
					3: 27.2	
					women (median):	
					1: 10.8	
					2: 16.0	
					3: 23.2	
Deleskog 2012 [77]	nested case-control study	Stockholm Diabetes Prevention Program	Sweden	*N* = 980 women, *N* = 1398 men	1: <18.52: 18.5–23.33: 23.3–28.44: >28.4	women:1: Ref2: 0.89 (0.59–1.35)3: 0.72 (0.47–1.11)4: 0.82 (0.53–1.28)
						men:1: Ref2: 0.75 (0.53–1.07)3: 0.81 (0.57–1.15)4: 0.80 (0.56–1.14)
Forouhi 2012 [80]	nested case-cohort study	Prospective Investigation into Cancer (EPIC)-Norfolk study	United Kingdom	*N* = 1447	1: <19.6	1: Ref
					2: 19.6–25.4	2: 0.66 (0.45–0.97)
					3: 25.5–32.1	3: 0.53 (0.34–0.82)
					4: >32.1	4: 0.50 (0.32–0.76)
Forouhi 2008/2012 [80,92]	Prospective study	Medical Research Council (MRC) Ely cohort	European Origin adults	Cases: 37 Non-cases: 740	1: <10.0	OR = 0.69 (0.17, 2.91), highest vs.lowest quartile
					2: 10.0–20.0	
					3: 20.1–30.0	
					4: ≥30.0	
Gonzalez-Molero 2012 [79]	cohort study	Population-based cohort from Andalusia	Spain	*N* = 1139	25th percentile:	1: Ref2: 0.17 (0.05–0.61)
					1: <18.5	
					2: ≥18.5	
Gorham 2012 [81]	nested case-cohort study	US Military service members (US Department of Defense, serological surveillance program)	USA	cases: 1000 controls: 1000	1: < 17.2	1: 3.5 (2.0–6.0)
					2: 17.2–23.6	2: 2.5 (1.5–4.2)
					3: 24.0–30.8	3: 0.8 (0.4–1.4)
					4: 31.3–39.7	4: 1.1 (0.6–2.8)
					5: ≥40.1	5: Ref
Husemoen 2012 [75]	cohort study	Inter99 study	Denmark	Baseline *N* = 6405 Follow-Up, *N* = 4296	1: <10.0	1: 1.65 (0.75–3.63)
					2: ≥10.0–20.0	2: 1.43 (0.73–2.80)
					3: ≥20.1–30.0	3: 1.25 (0.62–2.52)
					4: ≥30.0	4: Ref
Husemoen 2012 [74]	cohort study	MONICA 10 population	Denmark	*N* = 2571	1: <10.0	1: 1.42 (0.66–3.11)
					2: ≥ 10.0–20.0	2: 1.48 (1.04–2.12)
					3: ≥ 20.0–30.0	3: 1.30 (0.93–1.82)
					4: ≥30.0	4: Ref
Pilz 2012 [76]	cohort study	Hoorn study	The Netherlands	*N* = 351	1: <20.0	1: 2.15 (0.50–9.18)
					2: ≥20.0–<30.0	2: 1.64 (0.41–6.52)
					3: ≥30.0	3: Ref
Pittas 2012 [78]	cohort study	Diabetes Prevention Program (DPP)	USA	*N* = 2002	1: <12	1: Ref
					2: 12–19.9	2: 0.89 (0.81–0.97)
					3: 20–29.9	3: 0.76 (0.61–0.94)
					4: 30–49.9	4: 0.63(0.44–0.90)
					5: ≥50	5: 0.40 (0.230–0.81)
Afzal 2013 [55]	cohort study	Copenhagen City Heart Study	Denmark	*N* = 9841	1: <5	1: 1.22 (0.85–1.74)
					2: 5–9.9	2: 1.30 (1.06–1.59)
					3: 10–19.9	3: 1.22 (1.03–1.44)
					4: ≥20	4: Ref
Buijsse 2013 [82]	nested case-cohort study	German arm of the European Prospective Investigation into Cancer and Nutrition (EPIC)	Germany	*N* = 2121 participants *N* = 1572 incident cases of T2D	1: 2.6–12.0	1: Ref
					2: 12.0–15.8	2: 0.70 (0.52, 0.94)
					3: 15.9–20.1	3: 0.67 (0.50, 0.90)
					4: 20.2–25.4	4: 0.80 (0.59, 1.09)
					5: 25.4–119.0	5: 0.86 (0.62, 1.19)
Lim 2013 [83]	prospective study	Routine physical check at the Seoul National University Bundang Hospital (SNUBH)	Korea	*N* = 1080	1: <10	1: 3.23 (1.66–6.30)
					2: 10–19.9	2: 2.06 (1.22–3.49)
					3: ≥20.0	3: Ref
Schöttker 2013 [85]	cohort study	ESTHER cohort study	Germany	*N* = 7791	Median	
					1: 11.8	1: 1.17 (0.97–1.40)
					2: 14.8	2: 1.10 (0.92–1.31)
					3: 18.5	3: Ref
					4: 23.3	4: Ref
					5: 33.1	5: Ref
Tohidi 2013 [86]	nested case–control study	Tehran Lipid and Glucose Study	Iran	cases: 191 non-cases: 570	1: 2.82–11.02	1: Ref
					2: 11.03–21.80	2: 0.54 (0.29–1.00)
					3: ≥21.82	3: 0.40 (0.22–0.75)
Schafer 2014 [84]	prospective cohort study	Study of Osteoporotic Fractures	USA	*N* = 5463	1: 5–16	1: Ref
					2: 17–22	2: 1.09 (0.81–1.46)
					3: 23–28	3: 0.91 (0.66–1.26)
					4: 29–185	4: 0.92 (0.67–1.27)
Veronese 2014 [87]	population-based cohort study	Progetto Veneto Anziani (Pro.V.A.) Study	Italy	*N* = 2227	1: ≤10.0	1: 1.37 (0.87–2.16)
					2: 10.0–20.0	2: 1.44 (0.95–1.98)
					3: 20.0–30.0	3: 1.05 (0.76 –1.45)
					4: ≥30.0	4: Ref
Pham 2015 [89]	longitudinal study	Preventive health program of the Pure North S’Energy Foundation (PN)	Canada	*N* = 5730	1: <20.0	1: Ref (Risk for insulin resistance)
					2: 20.0–<30.0	2: 0.86 (0.67–1.11)
					3: 30.0–<40.1	3: 0.77 (0.58–1.04)
					4: 40.1–<50.1	4: 0.72 (0.52–1.00)
					5: ≥50.1	5: 0.68 (0.47–0.99)
Reis 2015 [88]	nested prospective cohort study	Atherosclerosis Risk in Communities (ARIC) Study	USA	*N* = 10222	1: 0.6–17.7	1: 1.37 (1.05, 1.80)
					2: 17.7–22.4	2: 1.22 (0.94, 1.58)
					3: 22.4–26.6	3: 1.03 (0.79, 1.34)
					4: 26.6–31.6	4: 1.33 (1.03, 1.71)
					5: 31.7–109.3	5: Ref
Napoli 2016 [90]	prospective cohort study	Multisite Osteoporotic Fractures in Men (MrOS) Study	USA	*N* = 1939	1: 3.13–20.89	1: Ref
					2: 20.90–25.63	2: 1.43 (0.89–2.30)
					3: 25.64–30.59	3: 1.62 (0.99–2.64)
					4: 30.60–74.77	4: 1.07 (0.61–1.89)
Le Fur 2016 [91]	prospective cohort study	Patients after renal transplantation	France	*N* = 444	1: <10	1: 2.41 (1.01–5.75)
					2: ≥10–<30	2: 1.22 (0.56–2.66)
					3: ≥30	3: Ref

* Ref: reference category; Conversion factor: nmol/L = ng/mL × 2.496.
